# Identification and characterization of regulatory elements in the promoter of *ACVR1*, the gene mutated in Fibrodysplasia Ossificans Progressiva

**DOI:** 10.1186/1750-1172-8-145

**Published:** 2013-09-18

**Authors:** Francesca Giacopelli, Serena Cappato, Laura Tonachini, Marzia Mura, Simona Di Lascio, Diego Fornasari, Roberto Ravazzolo, Renata Bocciardi

**Affiliations:** 1 Department of Neurosciences, Rehabilitation, Ophthalmogy, Genetics, Maternal and Child Health and CEBR, Università degli Studi di Genova, Genova, Italy; 2 Department of Medical Biotechnology and Translational Medicine, Università degli Studi di Milano, Milano, Italy; 3 CNR-Institute of Neuroscience, Milano, Italy; 4 Istituto Giannina Gaslini, Medical Genetics Unit, Genova, Italy

**Keywords:** ACVR1, Fibrodysplasia ossificans progressiva (FOP), *ACVR1* promoter, Transcriptional regulation, BMP signaling

## Abstract

**Background:**

The *ACVR1* gene encodes a type I receptor for bone morphogenetic proteins (BMPs). Mutations in the *ACVR1* gene are associated with Fibrodysplasia Ossificans Progressiva (FOP), a rare and extremely disabling disorder characterized by congenital malformation of the great toes and progressive heterotopic endochondral ossification in muscles and other non-skeletal tissues. Several aspects of FOP pathophysiology are still poorly understood, including mechanisms regulating *ACVR1* expression. This work aimed to identify regulatory elements that control *ACVR1* gene transcription.

**Methods and results:**

We first characterized the structure and composition of human *ACVR1* gene transcripts by identifying the transcription start site, and then characterized a 2.9 kb upstream region. This region showed strong activating activity when tested by reporter gene assays in transfected cells. We identified specific elements within the 2.9 kb region that are important for transcription factor binding using deletion constructs, co-transfection experiments with plasmids expressing selected transcription factors, site-directed mutagenesis of consensus binding-site sequences, and by protein/DNA binding assays. We also characterized a GC-rich minimal promoter region containing binding sites for the Sp1 transcription factor.

**Conclusions:**

Our results showed that several transcription factors such as Egr-1, Egr-2, ZBTB7A/LRF, and Hey1, regulate the *ACVR1* promoter by binding to the -762/-308 region, which is essential to confer maximal transcriptional activity. The Sp1 transcription factor acts at the most proximal promoter segment upstream of the transcription start site. We observed significant differences in different cell types suggesting tissue specificity of transcriptional regulation. These findings provide novel insights into the molecular mechanisms that regulate expression of the *ACVR1* gene and that could be targets of new strategies for future therapeutic treatments.

## Background

Fibrodysplasia Ossificans Progressiva (FOP, OMIM 135100) is a rare and extremely disabling disorder characterized by congenital malformation of the great toes and progressive endochondral heterotopic ossification (HO) [1]. Its prevalence is approximately 1/2,000.000, without ethnic, racial, gender or geographic predilection of disease. HO affects connective tissues such as skeletal muscle, tendon, ligaments, fascia, and aponeuroses with an episodic postnatal course characterized by flare-up and remission phases [1]. It is noteworthy to underscore that FOP flare-ups culminating in HO are frequently induced by soft tissue injury, such as trauma, muscle fatigue, surgery, infections, intramuscular injections, preschool immunizations; in other cases, FOP HO appears to initiate spontaneously without any apparent trigger [1].

Histological analyses of early FOP lesions from patients and animal models, show a first phase of strong local inflammation, with monocytes and lymphocytes infiltration and degeneration of skeletal muscle fibers. This is followed by a second phase characterized by a fibro-proliferative response and angiogenesis, ending with the formation of ectopic, but otherwise qualitatively normal, bone [2-4]. This evidence suggests that inflammation and immune mediated mechanisms play a crucial role in FOP disease progression and that activation of inflammatory pathways through the innate immune system could be relevant for postnatal flare-ups [3].

The molecular defect responsible for FOP is a heterozygous mutation in the *ACVR1* gene encoding the ALK2 Activin A type I receptor, a receptor for bone morphogenetic proteins (BMPs) [5]. Although most cases are sporadic (*de novo* mutations), a small number of inherited FOP cases show germline transmission in an autosomal dominant pattern [5]. The mutation that causes FOP moderately over-activates the BMP pathway in the absence of BMP binding [6-10], yet allows human embryonic development to occur relatively unimpaired with only mild skeletal effects, most typically malformation of great toes.

The majority of FOP patients are heterozygous for a recurrent activating mutation (c.617G>A; p.R206H) in the cytoplasmic GS (glycine-serine rich) domain of the receptor protein, as first reported by Shore and colleagues [5]. Other less frequent heterozygous *ACVR1* missense mutations in highly conserved amino acids located near the GS regulatory region or within the kinase domain, have also been described [11-16].

A very critical and still poorly understood aspect of the pathogenic mechanism of FOP is the nature of initial stimuli that trigger flare-up episodes to result in subsequent ossification in patients with the gene mutation (see reviews [2,3]). A plausible hypothesis is that the moderate hyper-activity of BMP signaling caused by the mutated receptor does not cause significant consequences under basal *in vivo* conditions in connective tissues. However, such a mutation would predispose the cells to respond to changes in the local tissue environment, such as inflammatory processes after tissue injury or other stimuli, by forming extra-skeletal bone. This hypothesis is consistent with quiescent periods that are observed in patients between active episodes of heterotopic bone formation.

Up to now, no effective and specific treatment is available and the only prevention is prophylactic measures against falls, infections, and soft tissue injuries [1]. The discovery of mutations in the *ACVR1* gene has opened the way to identify and characterize cellular and molecular mechanisms that can become targets for therapeutic control of heterotopic ossification in FOP.

While numerous articles and reviews about FOP and the BMP signaling pathway have been published, our knowledge of how *ACVR1* is regulated at different levels and how *ACVR1* transcription responds to changing cell environments under developmental, physiological or pathological conditions is still limited. For this reason, we conducted a characterization of the genomic region containing the *ACVR1* gene promoter in order to identify mechanisms and factors that regulate its expression at the transcriptional level. A previously published article described a regulatory region located upstream of the ATG start codon that was defined as the *ACVR1*-*ALK2* promoter by the authors [17]. However, the study did not consider the extended 5’UTR region shown in GenBank annotations for the *ACVR1* gene. This extended 5’UTR includes untranslated exons and long intronic sequences that predict a gene promoter region at ~78 kb upstream of the sequences described by Yao and coworkers [17].

In this study, we describe the characterization of a 2.9 kb *ACVR1* promoter region with respect to its transcriptional start site and show that a number of transcription factors play an important role in regulating *ACVR1* transcription. These data have potential to inform future studies regarding the regulation and function of the *ACVR1* gene in various processes as well as providing novel targets for future therapeutic approaches for FOP disease.

## Materials and methods

### Reagents, vectors and antibodies

Human recombinant BMP2 was purchased from R&D Systems (Minneapolis, MN). The following constructs were obtained as kind gifts from the indicated person/group: IdI-BMP-Responsive Element/Luciferase expression vector (BRE-Luc) from Dr Peter Ten Dijke; pCMV-Sp1 from Dr Francesco Ramirez; Egr-2 from Dr Marco Musso and Egr-1 from Dr Patrick Charnay. Expression vectors carrying the cDNA of Hey1 (isoform 1, v1, RefSeq NM_012258.2, SC115467; and isoform 2, v2, RefSeq NM_00104070.1, SC311179) and ZBTB7A (RefSeq NM_015898.2, SC114581) transcription factors were purchased from OriGene (TrueClone, OriGene Technology, Inc., Rockville, MD) as ready-to-transfect DNA.

Antibodies directed against Sp-1 (sc-59), Egr-1 (sc-189) and Egr-2 (sc-20690 and sc-20450) were obtained from Santa Cruz Biotechnology Inc.

### Bioinformatic analysis

Bioinformatic analysis of the genomic region at the 5' end of the *ACVR1* gene was performed using web-based software programs. Information on ESTs, positions of conserved binding sites for transcription factors, and data from the ENCODE project were obtained from the corresponding tracks of the UCSC Genome Browser in a window spanning a 2913 bp genomic region (chr2:158,732,343-158,735,255, UCSC Genome Browser, Feb2009 GRCh37/hg19 Assembly). Analysis of the GC nucleotide content used the CpG-plot tool at the EMBL-EBI European Bioinformatics Institute, [http://www.ebi.ac.uk/Tools/emboss/cpgplot].

The conservation profile was obtained with the tools for comparative genomics of the VISTA Genome Browser http://pipeline.lbl.gov/cgi-bin/gateway2. The search for transcription factor binding sites in selected regions, was performed with MatInspector software [http://www.genomatix.de/solutions/genomatix-software-suite.html] [18].

### Plasmid construction

The 2.9 kb genomic segment of the *ACVR1* promoter was reconstructed from two partially overlapping PCR fragments generated with LTR-1F + Prom-3Frev (fragment 1) and Sph-1F067-F/Rev (fragment 2) oligonucleotide couples, and the PCR Extender Polymerase Mix (5Prime) using the protocol for GC-rich templates suggested by the manufacturer. The two fragments were subcloned into the pCR2.1 vector (TOPO-TA cloning kit, Invitrogen), and orientation of the inserts was checked by restriction digests. Insert 2, obtained by double restriction with Kpn-1 (present in the multiple cloning site of the pCR2.1 vector) and Bgl-II (unique restriction site present in the region of overlap between the two PCR fragments), was then inserted into the intermediate pCR2.1-fragment 1 plasmid prepared with same double restriction. Finally, the resulting complete 2.9 kb insert was subcloned in the Kpn-I site of the pGL3-Basic expression vector (Promega). This plasmid construct was also used as a template in PCR reactions designed to obtain fragments of the promoter progressively deleted at the 5' end, by combining specific forward oligonucleotides with a common reverse primer mapping at the beginning of the first 5'UTR *ACVR1* exon (see Table 1, for position, length and oligonucleotides used to generate the corresponding fragment; see Table 2 for oligonucleotides sequences). All the constructs were checked by restriction enzyme digestion and direct sequencing according to standard protocols.

**Table 1:** List of the reporter constructs used in this work

**Construct**	**Insert length (bp)**	**Position on chr2 (UCSC Feb2009/GRCh37/hg19)**	**Name of oligonucleotides used**
**pPrACVR1-2.9**	2913	158,732,343-158,735,255	see text
**pPrACVR1-1.4**	1420	158,732,343-158,733,762	see text
**pPrACVR1-1.2**	1190	158,732,343-158,733,532	1.2-F+067-F/r
**pPrACVR1-0.7**	794	158,732,343-158,733,136	0.7-F+067-F/r
**pPrACVR1-0.6**	613	158,732,343-158,732,955	0.6-F+067-F/r
**pPrACVR1-0.3**	339	158,732,343-158,732,681	0.3-F+067-F/r
**pPrACVR1-0.16**	163	158,732,343-158,732,505	0.16-F+067-F/r
**pPrACVR1-0.072**	104	158,732,343-158,732,446	0.072-F+067-F/r

**Table 2:** Names and sequences of the oligonucleoides used in the present work

**Name**	**Sequence of oligonucleotides used in this work**
LTR-1F	CAAGTACTGTTACAGGACCC
Sph-1F	TGCTCCCACCTACTCCATCC
Prom-3Frev	GCCATGGGAGCTTACAGTG
067-F/REV	GAGGCGGAGTGCGAGGCAGC
6R	CGAAGGCAGCTAACTGTATC
1.2-F	(AGCT*GGTACC*)AGCCCCTCCATGCTTATTTC
0.7-F	(AGCT*GGTACC*)GCTCATAGCTTCCAC
0.6-F	(AGCT*GGTACC*)GGAGGAAATGATGTGTTGCTG
0.3-F	(AGCT*GGTACC*)GAAGTTTATTCTGCCC
0.16-F	(AGCT*GGTACC*)GCTGCAGCCACCGCAG
0.072-F	(AGCT*GGTACC*)CCGCAGAGTTCC
	**Oligonucleotides for site**-**directed mutagenesis**
Fr072mut-F	CACCGCCCCG**AA**CCG**AA**CCGCGCCG**AA**CCGCGCCGCG
Fr072mut-R	CGCGGCGCGG**TT**CGGCGCGG**TT**CGG**TT**CGGGGCGGTG
	**Oligonucleotides for EMSA**
GC-emsa F	GCCCCGCCCCGCCCCGCGCCGCCCCGCG
GC-emsa R	GGCGCGGGGCGGCGCGGGGCGGGGCGGGG
GCmut-emsa F	GCCCCGAACCGAACCGCGCCGAACCGCG
GCmut-emsa R	GGCGCGGTTCGGCGCGGTTCGGTTCGGGG

(agct*ggtacc*) Extrabases and Kpn-1 recognition site (*italic*) to facilitate PCR products subcloning in the pGL3-basic Luciferase expression vector.

### RNA extraction and cDNA synthesis

Total RNA was extracted from cells using the RNeasy Mini Kit from Qiagen. RNA quantity was measured with Nanodrop Spectrophotometer (Thermoscientific), and first strand cDNA was synthesized from 200 ng of total RNA with the Advantage RT-for-PCR Kit (Becton Dickinson) according to the manufacturer's protocol.

### Quantitative reverse transcription PCR (RT-qPCR)

Expression of the endogenous *ACVR1* gene was evaluated by RT-qPCR using specific TaqMan Gene Expression Assay (Applied Biosystems) (Table 3). Samples were measured in triplicate and the data were normalized using reference genes such as β-Actin, GAPDH and β2-Microglobulin, depending on the cell line. qPCR data (generated using a BioRad IQ5 instrument) was analyzed with Bio-Rad iQ5 software for Gene Expression studies. Under the experimental conditions used, no effect of mithramycin treatment on expression of the references genes was observed.

**Table 3:** List of the probes for RT-qPCR experiments used in this work

**Gene**	** TaqMan Gene Expression Assay (Species and ID specification)**	
*ACVR1*/*Alk2*	*Homo sapiens*	Hs00153836_m1
*acvr1*/*alk2*	*Mus musculus*	Mm00431645_m1
*β*-*Actin*	*Homo sapiens*	Hs99999903_m1
*β*-*Actin*	*Mus musculus*	Mm00607939_1
*GADPH*	*Homo sapiens*	Hs99999905_m1
*gadph*	*Mus musculus*	Mm99999915_g1
*β2*-*Microglobulin*	*Homo sapiens*	Hs99999907_m1
*β2*-*microglobulin*	*Mus musculus*	Mm00437762_m1

### Cell culture and transfection

U2OS, HeLa, and C2C12 cells were from ATCC (ATCC-LGC Standards Partnership). ATDC5 cells were obtained from the Cell Bank of the Riken Bioresource Center, Japan. Cells were cultured as follows: C2C12 (mouse myoblast cells) in α-minimal essential medium (Invitrogen) supplemented with 10% fetal bovine serum (FBS, Invitrogen); U2OS (human osteosarcoma cells) cells in McCoy's 5a Medium (Invitrogen) supplemented with 10% FBS; HeLa cells in Eagle's Minimum Essential Medium (Invitrogen) supplemented with 10% FBS; ATDC5 (mouse chondrogenic cell line derived from teratocarcinoma) cells in DMEM: Ham's F12 (1:1) (Invitrogen) with 5% FBS. All culture media were supplemented with 2 mM glutamine and 100U/ml penicillin and 100 μg/ml streptomycin and cells were grown in a humidified atmosphere with 5% CO_2_.

For transfection experiments, cells were plated at 25000-30000 cells/well in 24-well plates in the cell complete medium for one day then transfected with Lipofectamine 2000 (Invitrogen).

Briefly, the transfection mixtures contained 50 μl OptiMEM (Invitrogen), 2 μl Lipofectamine 2000, 25 ng pRL-TK-Renilla (Promega) as a control of transfection efficiency, 400 ng promoter-Luciferase constructs and, as indicated, 400 ng of a specific transcription factor expression vector or empty vector as control. 24 hours after transfection, cells were lysed with 100 μl Passive Lysis Buffer (Promega) for 20 min and 20 μl of the obtained lysate was used to measure Luciferase activity with the Dual-Luciferase Assay kit (Dual-Luciferase® Reporter Assay System, Promega) according to the manufacturer's procedure. Luciferase activity was quantified using the GLOMAX Multi Detection System (Promega).

As indicated, 24 hrs after transfection, cells were cultured in depletion medium (without FBS and in presence of 0.1% BSA) and treated overnight with 100 ng/ml BMP2 (R&D Systems) or mithramcycin at 200 or 300 nM. The impact of mithramycin treatment on cell viability was evaluated by a fluorescence-based viability assay (CellTiter-Fluor™Cell Viability Assay, Promega) and no significant effect was found upon the experimental conditions used.

### Preparation of nuclear extracts and electrophoretic mobility shift assay (EMSA)

Nuclear extracts were prepared from subconfluent HeLa cells. Cells were washed with PBS then detached with PBS/1 mM EDTA, pelleted, washed once with PBS, resuspended in 5 packed cell volumes of hypotonic buffer (10 mM Hepes, pH 7.9, 1.5 mM MgCl_2_, 10 mM KCl, 0.5 mM DTT, 0.5 mM phenylmethylsulfonyl fluoride), and incubated for 10 min on ice. After centrifugation at 4°C, the cells were resuspended in 3 packed-cell volumes of the same buffer containing 0.05% Nonidet P-40 (NP-40) and Dounce homogenized. The nuclei were collected by low speed centrifugation at 400 g for 10 min, then washed once with the same buffer described above, without NP-40, and resuspended in high salt buffer (5 mM Hepes, pH 7.9, 26% glycerol, 1.5 mM MgCl_2_, 0.2 mM EDTA, 420 mM NaCl, 0.5 mM DTT, 0.5 mM phenylmethylsulfonylfluoride), incubated at 4°C for 30–60 min, and centrifuged at 21,000 *g* at 4°C for 40 min. The supernatant was aliquoted and stored at -70°C; protein content was evaluated by Bradford assay.

EMSA was performed as previously described [19]. Briefly, double-stranded oligonucleotides were labeled by filling-in the 5' ends with 3000 Ci/mmol [a-^32^P]dCTP and purified on G-25 Sephadex column (Roche Applied Science). All probes were 100% labeled. Reactions containing 5 μg of nuclear extract, 10 μl of 2X binding buffer (1X binding buffer: 20 mM Hepes, pH 7.9, 2 mM MgCl_2_, 4% Ficoll, 0.5 mM DTT), 2 μg of double-stranded poly(dI-dC) (Sigma-Aldrich, St.Louis, MO, USA), 100 mM final salt concentration (NaCl + KCl) were assembled in a final volume of 20 μl and preincubated for 30 min on ice; 10,000–20,000 cpm of the labeled probes (corresponding to 2 fmol) were added to each reaction. After 30 min on ice, the reactions were electrophoresed through TBE, 5% non-denaturing polyacrylamide gels at a constant voltage of 150 V. Competition experiments and supershifts used reactions preincubated with the appropriate amounts of cold oligonucleotide and antibody (anti-SP1, Upstate, Charlottesville, VA and Santa Cruz Biotechnology). The oligonucleotides used for EMSA are reported in Table 2. The Sp1 and EGR oligonucleotides were described previously [19]. All oligonucleotides were synthesized by TIB MolBiol (Roche).

### Statistical analysis

All luciferase reporter gene assays using the *ACVR1* promoter constructs were performed in triplicate and repeated independently at least twice (2-5 times). Experiments to evaluate *ACVR1* expression by RT-qPCR were perfomed in triplicate from at least two independent RNA extractions.

As indicated, the non-parametric Mann–Whitney Test (GraphPad InSTAT package) was applied to verify statistical significance of the observed variations, significant differences were given as *p* < 0.05^*^, *p* < 0.01^**^, or *p* < 0.001***.

## Results

### Characterization of the *ACVR1* promoter

To identify and characterize the *ACVR1* promoter sequence and RNA transcriptional start site, we conducted both a bioinformatic analysis of the genomic region located 5' upstream of the gene and an experimental approach through 5'RACE. Current GenBank annotation reports two different mRNA RefSeq (NM_001111067.2 and NM_001105.4) that consist of two untranslated exons preceding nine protein-coding exons. According to these observations, putative alternative 5'UTR sequences are combined to common protein-coding sequences that initiate in the third exon and extend over nine exons. 5'RACE experiments using cDNA from adult brain detected an *ACVR1* transcription start site (TSS) mapping between 244 and 237 bp upstream of the ATG starting codon relative to the NM_001111067.2 RefSeq (not shown). This TSS corresponds to the start of the most upstream untranslated exon, possibly representing the most abundant transcript in the analyzed tissue and differing in only five base pairs compared to GenBank NM_001111067.2.

Therefore, we started our experimental analysis by focusing on the genomic sequence upstream of this position and spanning around 2.9 kb (chr2:158,732,343-158,735,255, UCSC Genome Browser, Human Feb. 2009 (GRCh37/hg19)).

**Figure 1:**
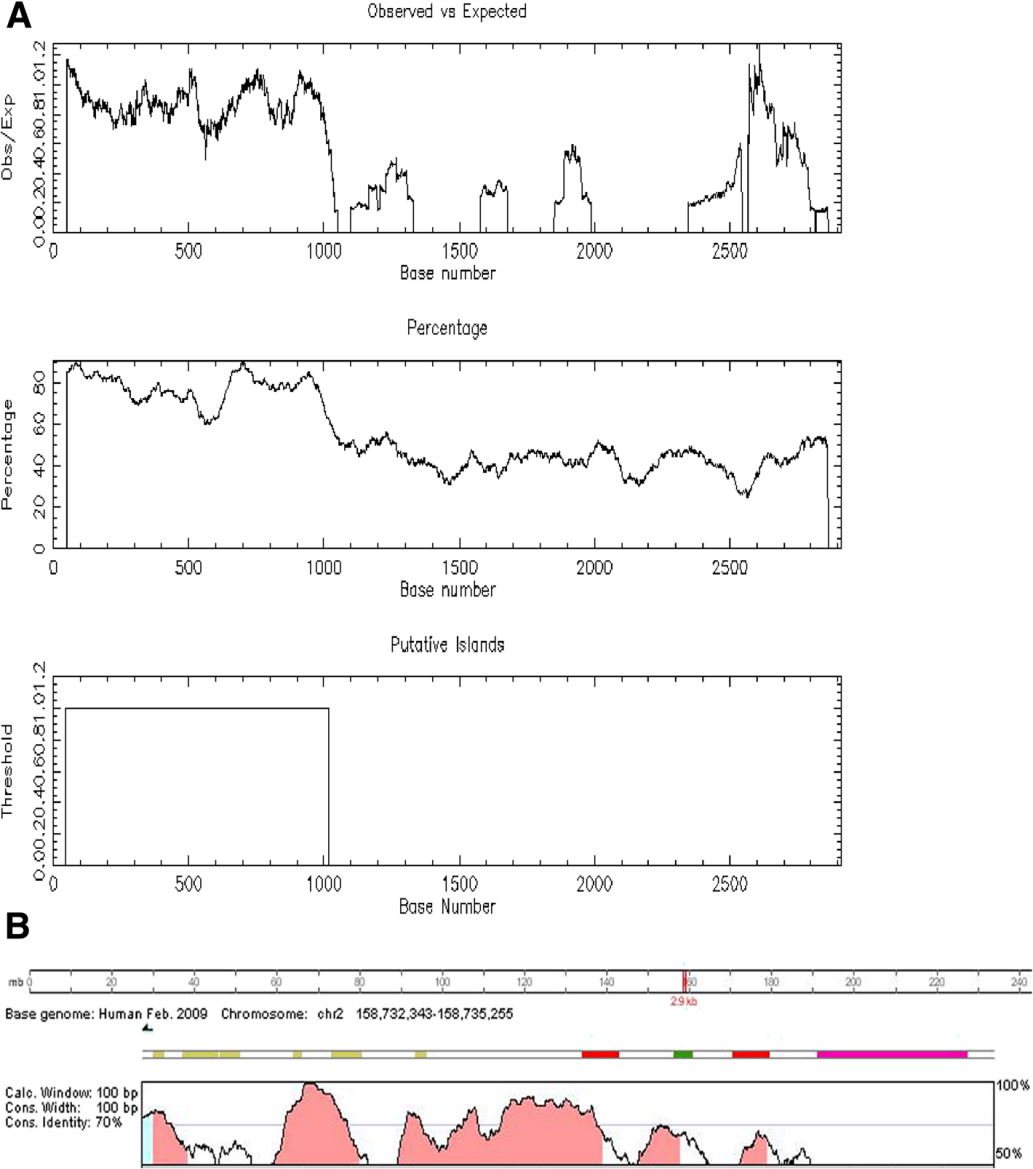
**Base composition and conservation of the human *ACVR*1 promoter. A)** CpG islands plot reporting the analysis of the GC-nucleotide content and structure of the *ACVR1* 2.9 kb genomic region upstream of the TSS using the CpG-plot tool at the EMBL-EBI European Bioinformatics Institut, [http://www.ebi.ac.uk/Tools/emboss/cpgplot]. Upper panel: plot of the ratio of the observed over the expected GC content. Middle panel: GC content expressed as percentage (minimum percentage required to identify the presence of a CpG islands is 50%). Lower panel: identification of a 975 bp CpG island upstream and proximal to the first 5'UTR exon of the *ACVR1* gene. **B)** Output of the comparative genomic analysis obtained with dedicated software available at the Vista Genome Browser (http://genome.lbl.gov/vista/index.shtml, tools for comparative analysis). Conserved sequences with a percentage of identity (vertical axis) that is higher than 70% are represented as red peaks (non coding sequences) and light blue peaks (UTR sequences, corresponding to the first UTR exon of the *ACVR1* gene according to the NM_001111067.2 RefSeq) and are shown relative to their position in the human genome (horizontal axis) compared to the mouse.

This region has 55% of GC nucleotides clustered in long stretches predominantly mapping to the proximal 1 kb segment, as indicated in the GC-plot reported in Figure 1A. No TATA-boxes are present. The region appeared well-conserved in mouse as shown by comparison analysis performed at the Vista Genome Browser (Figure 1B).

**Figure 2:**
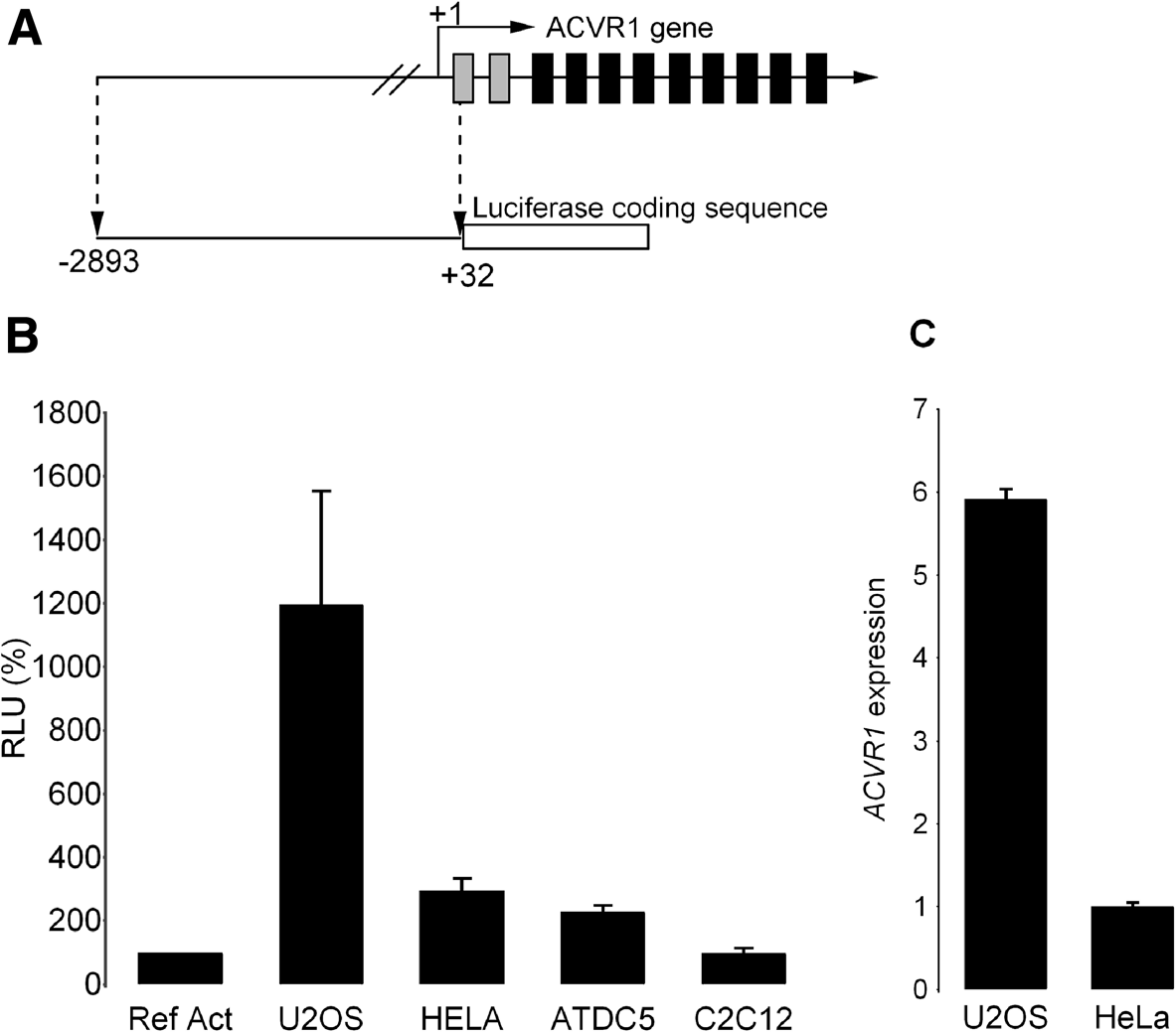
**Transcriptional activity of the 2.9 kb promoter region of human *ACVR1 *gene in different cell lines. A)** Schematic representation of the *ACVR1* genomic structure and of the reporter construct containing the 2.9 kb genomic fragment. The gene consists of eleven exons, two untranslated (grey rectangles) and nine protein coding exons (black rectangles). The 2.9 region upstream the TSS indicated as +1 was subcloned upstream the Luciferase coding sequence as described in the Materials and Methods (this reporter construct is referred as Pr-2.9). **B)** Results obtained by transient transfection of the Pr-2.9 construct in the indicated cell lines. Transcriptional activity is reported as relative to the activity of the pGL3-Promoter vector carrying the SV40 promoter as control (Ref Act, 100%, RLU, Relative Light Unit). **C)** mRNA levels of the endogenous *ACVR1* gene as assessed by RT-qPCR in U2OS and HeLa cells. *GAPDH* and *β2*-*Microglobulin* were used as Reference genes.

The entire 2.9 kb sequence was subcloned into the pGL3-basic Luciferase expression vector to generate the pPr-2.9 construct (Figure 2A). In order to determine whether this fragment was able to drive the expression of the Luciferase reporter gene, the plasmid was transfected into different cell lines (U2OS, HeLa, C2C12 and ATDC5). As shown in Figure 2B, this construct had strong promoter activity as compared to cells with the pGL3-Promoter vector carrying the SV40 promoter sequence. The level of transcriptional activity was different in the various cell lines, with the strongest activity in U2OS cells and the lowest in C2C12. In accordance, we found that the level of mRNA expressed from the endogenous *ACVR1* gene in U2OS and HeLa cells was proportional to promoter activity (Figure 2C).

**Figure 3:**
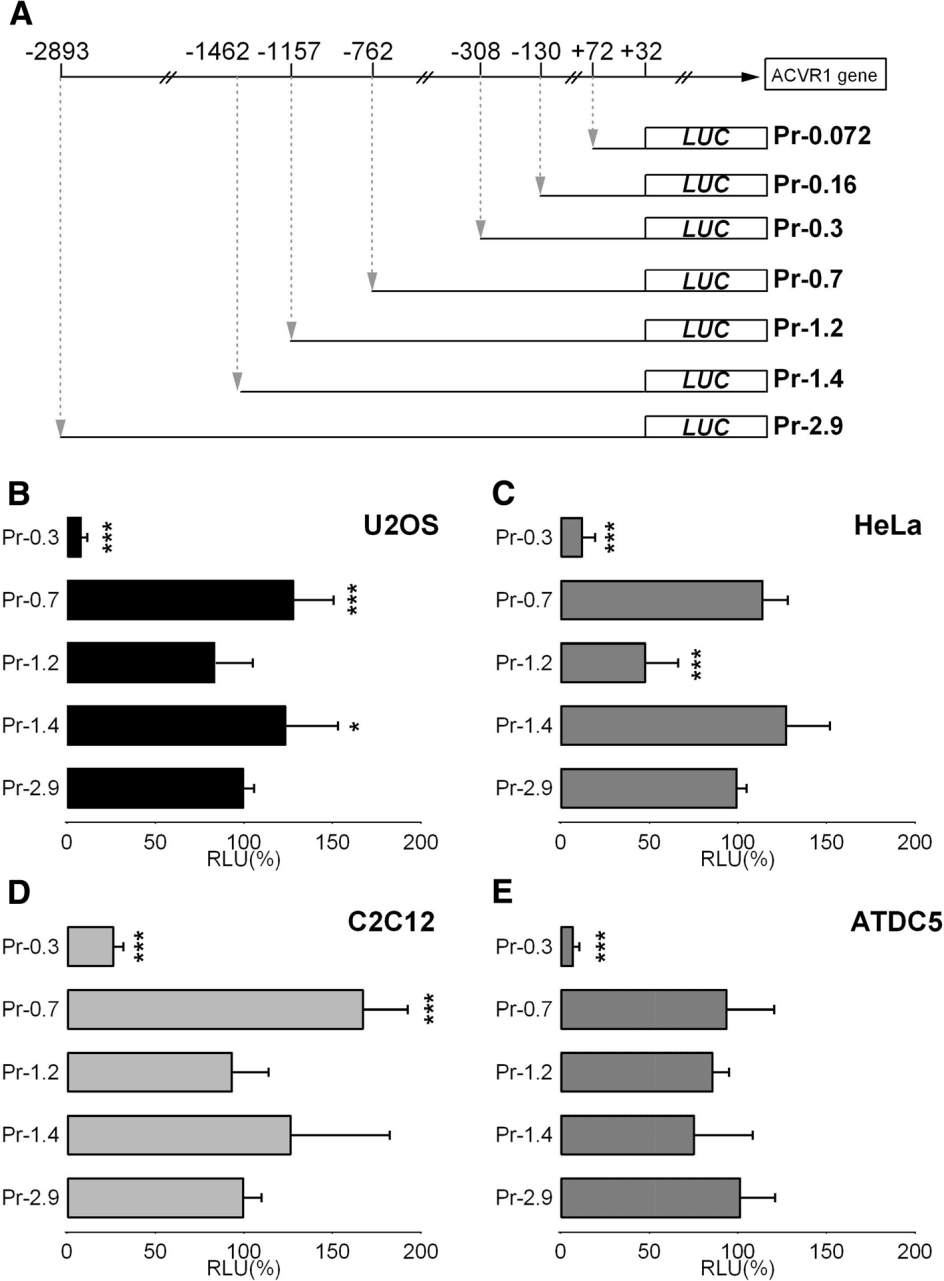
**Functional characterization of the 2.9 kb promoter region of human *ACVR1 *gene. A)** Schematic representation of the deletion constructs derived from the *ACVR1* 2.9 kb promoter region. These reporter constructs were transiently transfected in U2OS **(B)**, HeLa **(C)**, C2C12 **(D)** and ATDC5 cells **(E)**, together with the pRL-TK-Renilla plasmid as a control for transfection efficiency. The corresponding observed activities are shown as relative to the activity of the Pr-2.9 *ACVR1* promoter construct (100%). The data represent the mean ± SD (error bars) of five independent experiments conducted in triplicate, with *p* < 0.05^*^, *p* < 0.01^**^, or *p* < 0.001*** comparing the activity of each deletion construct with the 2.9 kb promoter.

Deletion constructs of the 2.9 kb region were generated in order to more specifically identify regulatory elements. Deletion regions were selected based on the human/mouse conservation profile, the presence of GC-clusters, and putative recognition sites for transcription factors, and the deletion constructs are schematically represented in Figure 3A.

All the generated constructs were transfected into multiple cell lines and their respective transcriptional activities are shown in Figure 3B relative to the activity driven by the pPr-2.9 plasmid containing the entire promoter region. The two longest deletion constructs (pPr-1.4 and pPr-1.2) showed similar promoter activity as the 2.9 kb fragment (pPr-2.9). Deletion of the region between position -1157 and -762 led to limited increase of the transcriptional activity, probably because of the removal of negative *cis*-acting elements functional in U2OS, HeLa and C2C12 cells. Deletion of the region between -762 and -308 caused a decrease in transcriptional activity in all the tested cell lines, suggesting the presence of functional elements important for promoter activity. The smallest pPr-0.3 construct still showed a clearly detectable activity. The pattern of transcriptional activity driven by the deletion constructs was similar in all the tested cell lines.

### Effects of Egr-1, Hey-1 and ZBTB7A/LRF/pokemon on *ACVR1* transcription

The observation that deletion of the genomic region comprised between -762 and -308 resulted in a strong reduction of the transcriptional activity driven by the *ACVR1* promoter, induced us to focus our functional analysis on this sequence.

Data obtained through the ENCODE Project (Encyclopedia of DNA Elements) provides experimental ChIP-seq (deep sequencing after Chromatin Immuno-Precipitation) evidence of transcription factor binding at the genome-wide level. ENCODE data for the 2.9 kb region of the *ACVR1* promoter provided evidence of transcription factor binding, as shown in Additional file 1: Figure S1 (ENCODE data [20]). We selected some of these transcription factors for further *ACVR1* functional analysis based on their reported involvement in osteogenic processes [21-23], immune-mediated mechanisms [24-26], and cross talk among important pathways [27-29].

**Figure 4:**
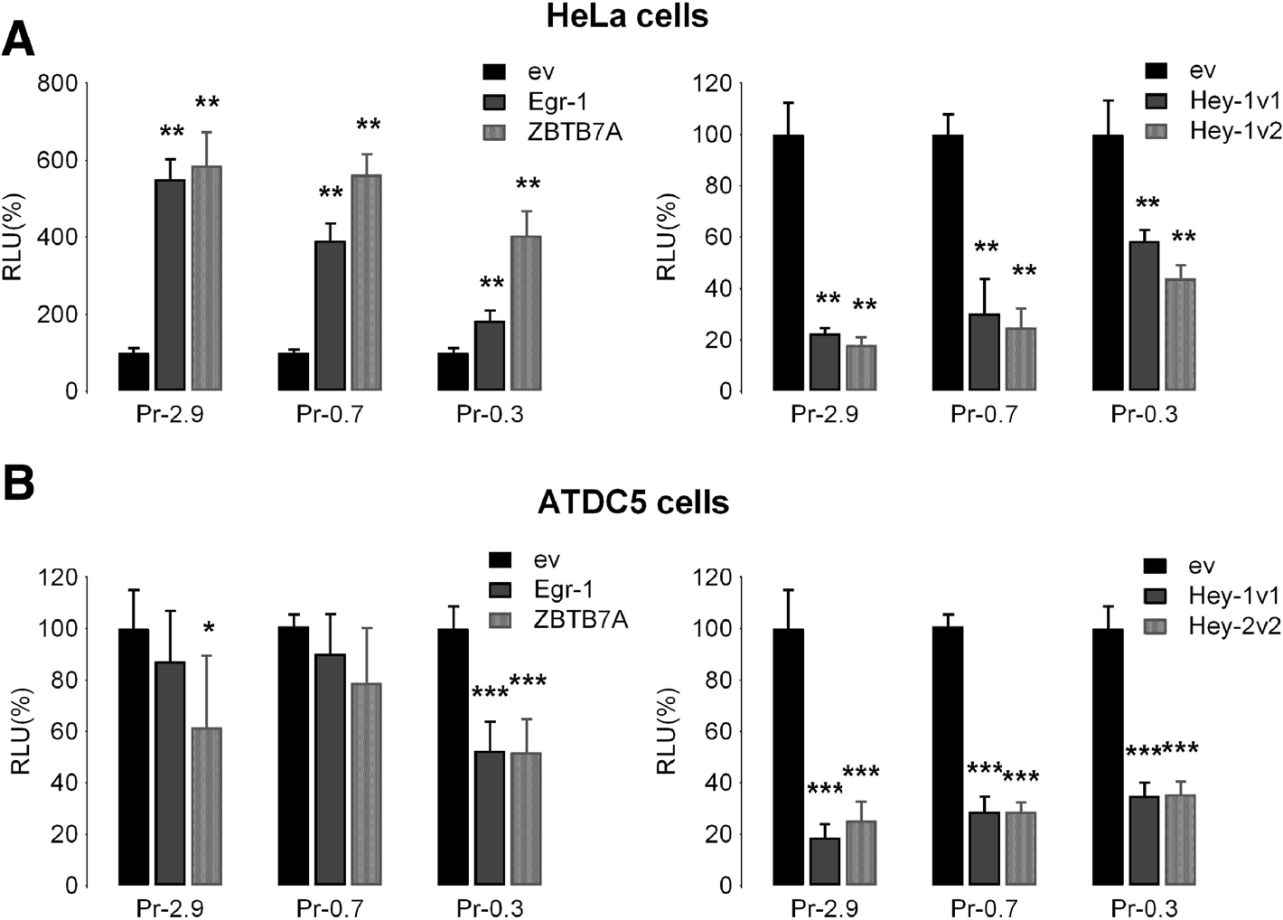
**Effects of Egr-1, Hey-1 and ZBTB7A/LRF/pokemon on *ACVR*1 promoter activity.** Expression vectors carrying the cDNA of Egr-1, Hey-1 or ZBTB7A/LRF/pokemon transcription factors were transiently transfected in the HeLa **(A)** and ATDC5 **(B)** cell lines together with the three reporter *ACVR1* promoter deletion constructs (Pr-2.9, Pr-0.7 and Pr-0.3) and the pRL-TK-Renilla plasmid as control for transfection efficiency. Detected activities are expressed relative to those of the same promoter construct co-transfected with empty expression vector considered as 100% (RLU, Relative Light Unit). The data represent the mean ± SD (error bars) of independent experiments (n=2 in HeLa cells, n=3 in ATDC5 cells) carried out in triplicate with *p* < 0.05^*^, *p* < 0.01^**^, or *p* < 0.001***. *ev*, empty vector, corresponding to the empty expression plasmid of the selected transcription factors. For the Hey-1 protein, two different cDNAs corresponding to two isoforms (indicated as v1 and v2) differing by four residues in the HLH domain were used.

Egr-1, Hey-1, and ZBTB7A cDNA expression vectors were co-transfected with the reporter constructs of selected deletion fragments of the *ACVR1* promoter (pPr-2.9, pPr-0.7 and pPr-0.3) into HeLa and ATDC5 cells representing, respectively, cell types not related and related to chondrogenesis/osteogenesis. Our results showed that the effect of these factors was dependent on cell type, at least for Egr-1 and ZBTB7A. As shown in Figure 4A, in HeLa cells both Egr-1 and ZBTB7A induced a strong activating effect on the transcriptional activity of the 2.9 kb *ACVR1* promoter. For Egr-1, the effect was progressively reduced with deletion constructs (Figure 4A, left panel), whereas ZBTB7A still exerted a stimulatory effect also on the smallest pPr-0.3 fragment. Consistently, the evidence provided by the ENCODE project indicates that binding sites for ZBTB7A are present in the genomic region contained in this short construct. However, in ATDC5 cells, the two proteins have little effect on the activity controlled by the whole *ACVR1* promoter and by the 700 bp-containing deletion construct. Interestingly, in these cells, ZBTB7A showed an inhibitory effect on the function of the smallest fragment tested (Figure 4B, left panel).

By contrast to Egr-1 and ZBTB7A, expression of both Hey-1 isoforms induced a decrease in the transcriptional activity driven by the *ACVR1* promoter (Figure 4, right panels), in both HeLa and ATDC5 cell lines, although with some quantitative differences between the two cell types. This effect was observed for all three promoter segments, as we might expect from the presence of binding sites in the -762/-308 region and also in the most proximal sequence of the promoter (Additional file 1: Figure S1).

### Characterization of a basal promoter region

As indicated above, the *ACVR1* promoter sequence does not contain a TATA box and is GC-rich, suggesting that the region proximal to the TSS might function as a basal promoter. To identify the region necessary for basal transcription of *ACVR1*, two additional deletion constructs were generated (see Figure 3A for a schematic representation of the constructs), pPr-0.16 and pPr-0.072, containing only 160 or 72 bp upstream of the TSS, respectively.

**Figure 5:**
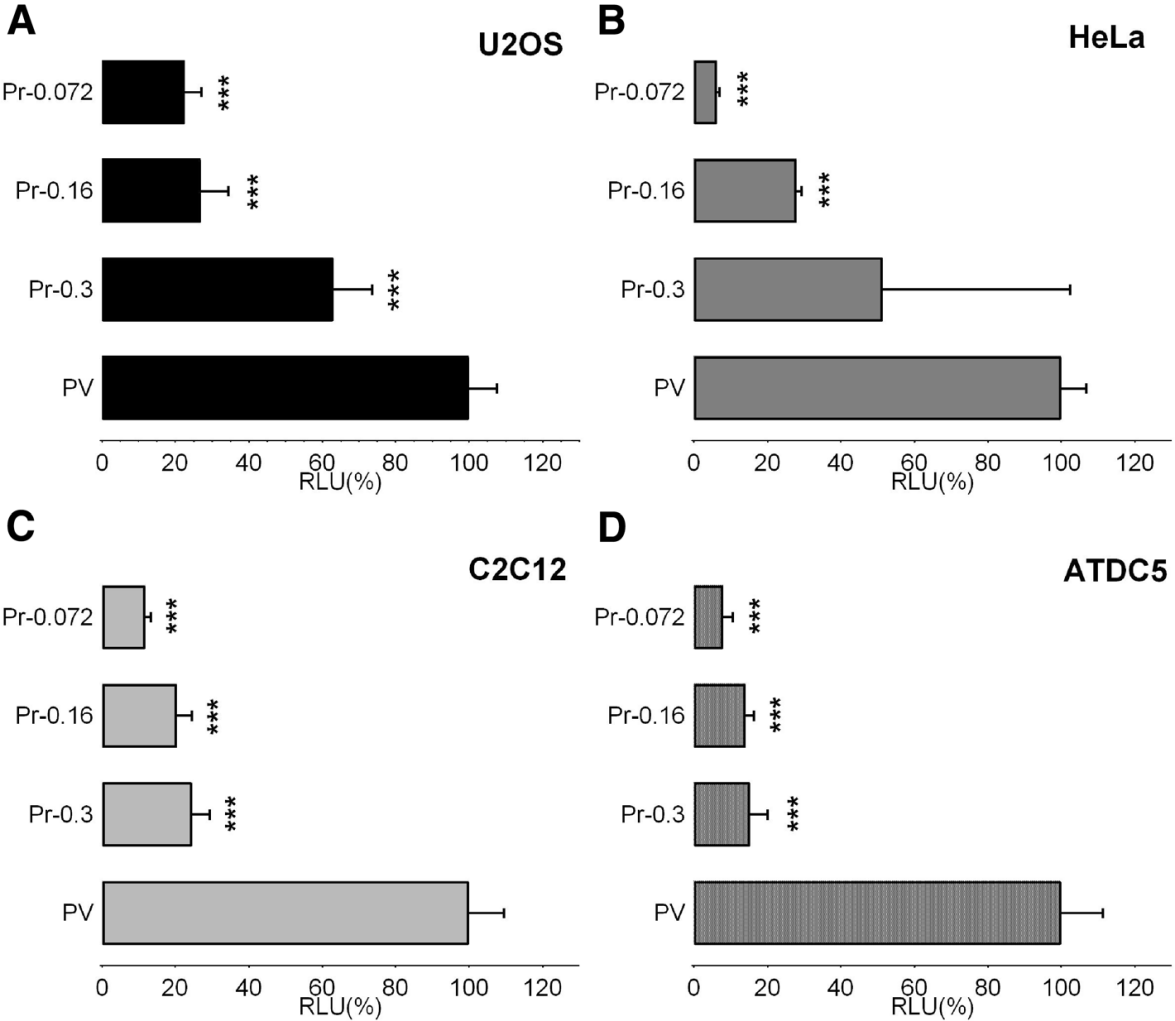
**Identification of the basal promoter.** Deletion constructs Pr-0.3, Pr-0.16, Pr-0.072 schematically represented in Figure 3A were transiently transfected into U2OS **(A)**, HeLa **(B)**, C2C12 **(C)**, ATDC5 **(D)** to characterize the basal promoter region. Results are expressed as relative to the activity of the pGL3-Promoter vector carrying the SV40 promoter region (PV, 100%). The data represent the mean ± SD (error bars) of independent experiments (3 ≥ n ≥ 5) carried out in triplicate with *p* < 0.05^*^, *p* < 0.01^**^, or *p* < 0.001***. comparing the activity of each deletion construct with that of the SV40 promoter (PV).

**Figure 6:**
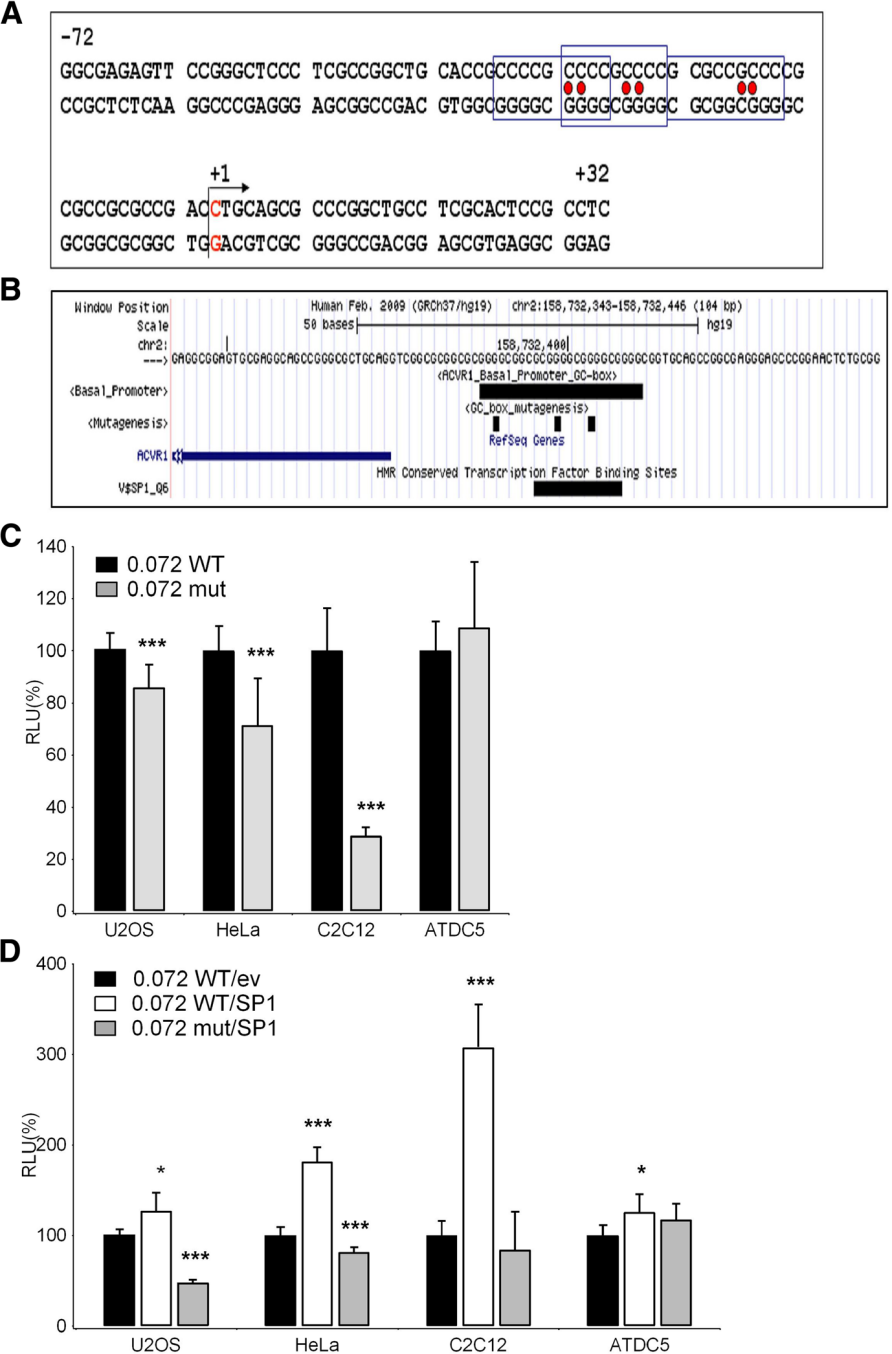
**Role of Sp1 in *ACVR1 *gene promoter regulation. A)** Nucleotide sequence of the basal promoter of the *ACVR*1 gene lacks TATA boxes and has high GC-nucleotide content. The putative Sp1 binding sites are indicated by boxes and nucleotides subjected to site-directed mutagenesis are indicated with red circles. The arrow indicates the TSS (+1). **B)***In silico* analysis of the region as it appears in the HMR Conserved Transcription Factor Binding Sites (TFBS conserved) track in the UCSC Genome Browser revealed a well-conserved putative recognition site for the Sp1 transcription factor. Custom tracks indicating the overlapping GC-boxes (ACVR1_Basal_Promoter_GC-Boxes) and the position of the mutagenized nucleotides (GC-boxes_mutagenesis) were added to the UCSC window and are shown. **C)** Both the wild-type (WT) and mutated Pr-0.072 constructs containing the 72 bp promoter region were transiently transfected into U2OS, HeLa, ATDC5, and C2C12 cell lines. Luciferase activities obtained with mutated constructs are expressed as a percentage of the activity of the wild-type (100%) in each cell line. **D)** The effect of Sp1 on basal promoter activity was tested by co-transfecting the Sp1 expression plasmid with the wild-type (WT) and mutated Pr-0.072 reporter constructs in the indicated cell lines. Luciferase activity is expressed as relative to the transcriptional activity of the Pr-0.072WT in cells transfected with the empty vector that was used to subclone the Sp1 cDNA (*ev*). The data represent the mean ± SD (error bars) of independent experiments (n=5 for C, n=3 for D) carried out in triplicate with *p*< 0.05^*^, *p*< 0.01^**^, or *p*< 0.001***.

Figure 5 shows that, after progressive deletion from -300 bp up to -72 bp, transcriptional activity was maintained in all transfected cell lines, although with quantitative differences. Nevertheless, the residual activity driven by the smallest fragment (pPr-0.072) was still significant, ranging from 6% to 20% depending on the cell lines used (Figure 5). Therefore, we have defined this 72 bp region as the basal promoter of the *ACVR1* gene. The region contains 87.5% GC (63 GC nucleotides out of 72) that are organized in overlapping GC-boxes (Figure 6A). This feature is a common finding in TATA-less gene promoters, and these sequence elements are likely to function as docking sites for GC-binding proteins.

An *in silico* search for transcription factors binding sites revealed that the region contains a well-conserved putative recognition site for the Sp1 transcription factor (Figure 6B), an established GC-binding protein able to regulate both basal and induced gene transcription working alone, or by recruiting other transcription factors (for a review see [30]).

We tested whether the Sp1 protein could influence the transcriptional activity driven by the 72 bp basal region by performing site-directed mutagenesis to destroy the overlapping GC boxes predicted to bind the protein (Figure 6A and 6B). As reported in Figure 6C, site-directed mutagenesis altering the GC-boxes affected the transcriptional activity of the *ACVR1* basal promoter in HeLa (reduced to 57% of the WT activity) and in C2C12 cells (29% of the WT activity) (Figure 6C). The effect was less evident in U2OS cells (86% of activity was maintained) and no effect was detectable in ATDC5 cells (Figure 6C). We then co-transfected cells with the pPr-0.072 construct (wild type or mutant) and an expression vector carrying the Sp1 cDNA (pCMV-Sp1) or the corresponding CMV empty vector as a control. Over-expression of Sp1 increased the transcriptional activity of the basal promoter, although to different levels in the various cell lines (Figure 6D). This induction was impaired by mutagenesis of the GC-boxes, except for the ATDC5 cell line.

**Figure 7:**
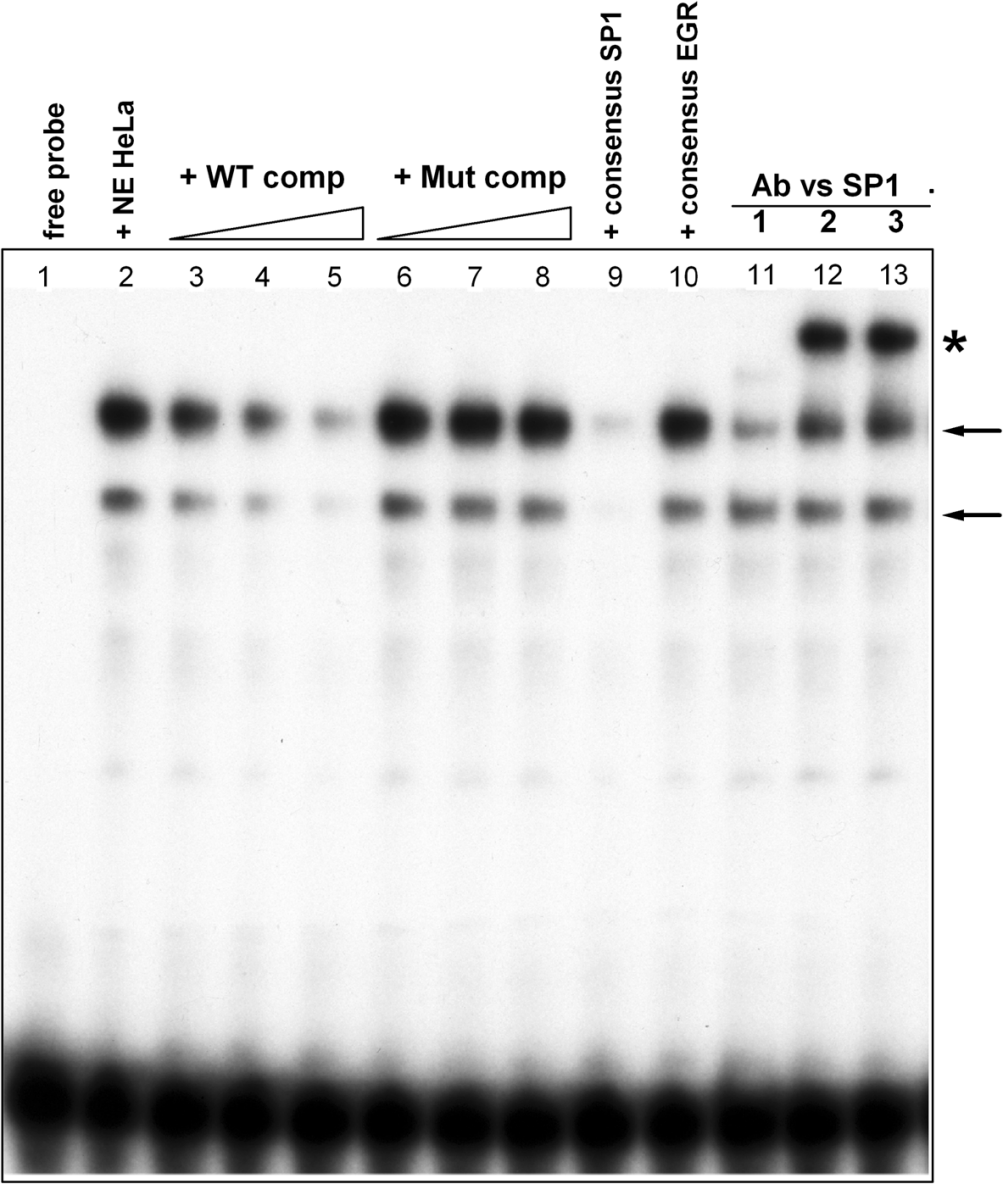
**Analysis of Sp1 binding to the *ACVR1 *72-bp promoter region.** The Sp1 transcription factor binds the *ACVR1* basal promoter as assessed by EMSA experiments with HeLa nuclear extracts and a double-stranded probe spanning the GC-boxes contained in the fragment (position -38 to -9). *Lane 1*, Free probe; *lane 2*, probe incubated with HeLa nuclear extracts (NE); *lanes 3 to 5*, DNA-protein complexes (arrows) competed with the cold wild-type probe (50-, 100-, or 200-fold molar excess, respectively); *lanes 4 to 8*, DNA-protein complexes competed with the cold mutated probe (50-, 100-, or 200-fold molar excess, respectively); *lanes 9 and 10*, DNA-proteins complexes incubated with probes containing canonical consensus sites for Sp1 and Egr-family factors respectively; *lanes 11 to 13*, super-shift analyses of the DNA-protein complexes with three different specific antibodies against Sp1. *, retarded complexes recognized by anti-Sp1 antibodies.

Consistently, treatment of U2OS and C2C12 cells with mithramycin A, an antibiotic frequently used as an Sp1 inhibitor [31], led to a dose-dependent decrease in the expression of endogenous *ACVR1* gene, as assessed by RT-qPCR in the U2OS and C2C12 cell lines (Additional file 2: Figure S2, supplementary material). To complete our analysis on the involvement of the Sp1 transcription factor in *ACVR1* basal promoter regulation, we carried out an Electrophoretic Mobility Shift Assay (EMSA) by using a 30 bp double-stranded probe overlapping the GC boxes with nuclear extracts from different cell lines. In Figure 7, results obtained with HeLa nuclear extracts are shown. Specific DNA/Sp1 protein complexes were detected (Figure 7, arrows) which were efficiently competed by the unlabeled probe (Figure 7, +WT comp), but not by a cold probe carrying the same mutated residues as in the transfected constructs (+Mut comp). Moreover, the observed complexes were further shifted by the presence of antibodies against Sp1, thus identifying Sp1 as able to bind sequences in this genomic region (Figure 7, asterisk).

Besides Sp1, other factors such as Egr-1 and 2 proteins are known to bind GC-rich recognition sequences. We co-transfected cells with the pPr-0.072 wild-type and expression vectors carrying the Egr-1 or Egr-2 cDNAs or the corresponding empty vector. As shown in Additional file 3: Figure S3, Egr-1 expression induced a decrease in the *ACVR1* promoter-Luciferase activity in U2OS, C2C12 and ATDC5 cells at comparable levels. No effect was observed in HeLa cells. Interestingly, a more cell type-dependent effect was detectable upon expression of Egr-2 proteins. As reported in Additional file 3: Figure S3, no or little effect was observed in U2OS, whereas a clear activating effect was observed in the other cell lines, particularly evident in HeLa and C2C12.

**Figure 8:**
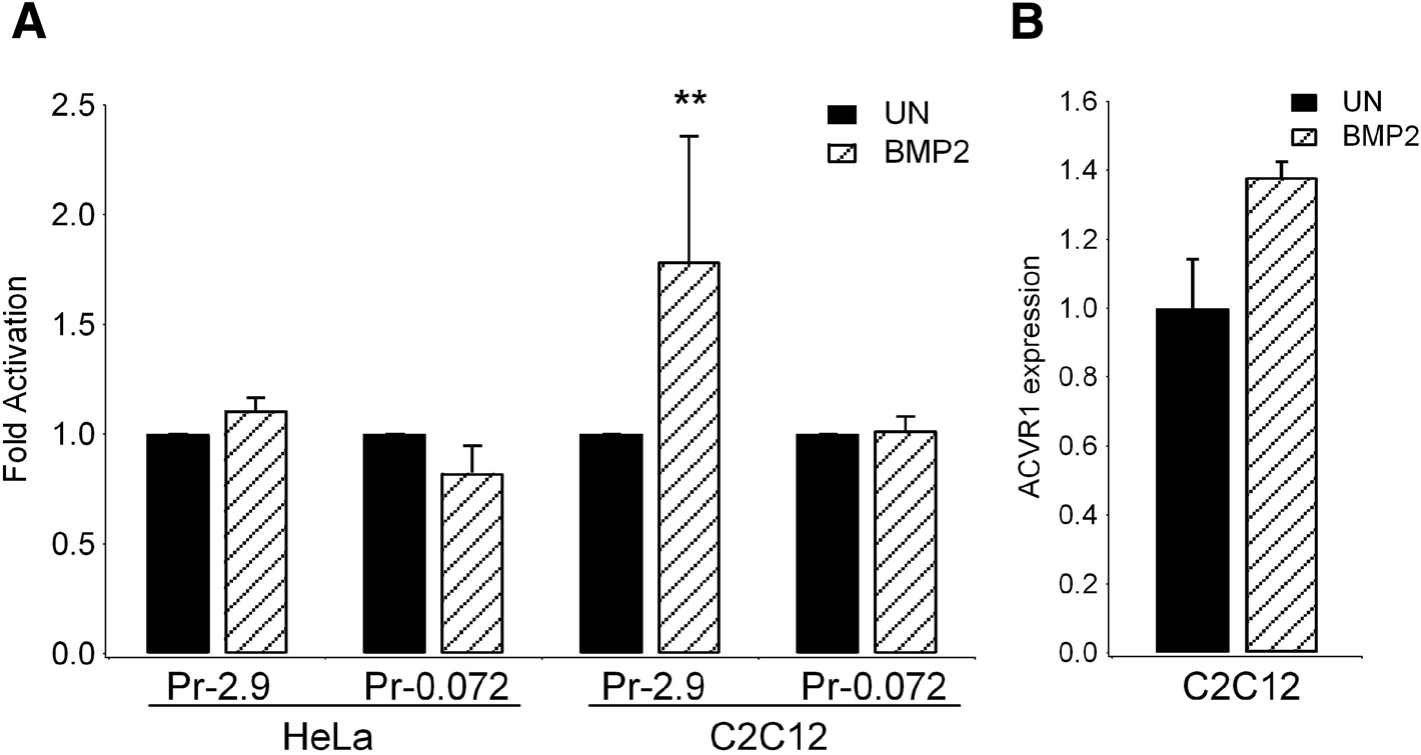
**Effect of BMP2 on *ACVR*1 promoter activity. A)** HeLa and C2C12 cells were transfected with Pr-2.9 and Pr-0.072 reporter constructs and Luciferase expression was determined in the presence or absence of 100 ng/ml BMP2. Results are expressed as fold activation relative to the activity by the same promoter construct in absence of BMP2 (UN, untreated), with *p*< 0.05^*^, *p*< 0.01^**^, or *p*< 0.001***. **B)***ACVR1* mRNA expression levels were detected by RT-qPCR in C2C12 cells upon treatment with BMP2. Values were normalized to the expression level of *GAPDH* and *β*-*Actin* genes.

An additional regulatory element, one that would be particularly important in the promoter of a gene encoding a BMP receptor, is the BMP Responsive Element (BRE) [32]. Although *in silico* search for such element did not predict its presence in the above mentioned promoter segment, these regulatory elements are characterized by a GC-rich sequence. Therefore, we wanted to verify if the Luciferase construct with the 72 bp basal promoter sequence is responsive to BMP ligands. Transfection experiments in HeLa and C2C12 cells treated with BMP2 (Figure 8) showed no effect on the 72 bp promoter region in both cell types. However, the constructs containing the 2.9 kb region were responsive to BMP2 treatment in C2C12 cells, although not in HeLa. We also observed an increase in *ACVR1* mRNA in C2C12 cells after treatment with BMP2. In agreement with the experimental data, *in silico* analysis predicts the presence of two BRE elements in the distal half of the gene (position -1188/-1183 and -657/-652, respectively).

## Discussion

In the present study, we provided the first characterization of the *ACVR1* gene promoter region. This gene is mutated in patients affected by FOP, causing a hyper-activation of the BMP SMAD-dependent signaling pathway. The pathogenic events resulting in heterotopic ossification in FOP are associated with mechanisms related to inflammatory stimuli that trigger osteogenic differentiation in pluripotent progenitors in postnatal life. It is conceivable that these mechanisms could be targeted to some levels of BMP signaling pathways thus providing strategies to control the most devastating effects of this genetically-based disorder in extra-skeletal tissues.

The discovery of *ACVR1* as the gene responsible for FOP has opened the way to treatment discovery efforts, considering that the identification of a molecular target related to the disease is the very first step of a drug development process. Elements and factors controlling regulation of gene and protein expression can be considered as "sensitive nodes" (as defined in [33]) and thus may become therapeutic targets. With this objective in mind, we considered that research on transcriptional mechanisms controlling expression of the *ACVR1* gene expression might provide significant contributions to identifying sensitive molecular targets.

To our knowledge, no previous characterization of the *ACVR1* promoter has been described and little information about the transcriptional regulation of the gene has been generated, with the exception of a study reporting a regulatory element upstream of the translation initiation codon [17]. *ACVR1* has a transcribed and untranslated region 5' to the translational start site that is quite complicated in term of untranslated exons and alternative splicing (unpublished observations and GenBank annotations). Thus a first requirement was to identify a TSS by 5'RACE experiments; we identified a major TSS that is located at the 5'end of the first untranslated exon, at least for the cells utilized for mRNA extraction. Given the predicted complexity of the *ACVR1* 5'UTR, this result, although confirming the presence of a TSS starting upstream of UTR exons, does not exclude other alternative TSSs that might represent low abundance transcripts or transcripts with cell type specificity different from the site we have identified.

This result allowed us to explore the sequence upstream of the identified TSS for base composition, conservation across species, and putative transcription factor binding sites.

We cloned a region from -2900 bp to +35 relative to the TSS (considered as the +1 nucleotide), into a Luciferase expression reporter plasmid. This region showed a robust ability to drive reporter vector expression, suggesting that it represents the gene transcriptional regulatory region. One first observation was differences in activity in various cell lines, with the highest activity in U2OS osteosarcoma cells, consistent with a high expression of *ACVR1* mRNA in this cell line, and suggesting the presence of factors that target the regulatory sequence in optimal condition compared to other cell types. Other cell types, however, are also active in regulating the *ACVR1* promoter relative to the reference activity of the SV40 promoter in the pGL3-Promoter vector.

The analysis of deletion constructs revealed the presence of positive and negative regulatory elements. Removal of the region between -1462 and 1158 showed different effects among cell types, such as significant reduction of activity in HeLa cells, that deserves further analysis for more detailed interpretation. In the case of ATDC5 and C2C12 cells, two cell types that are able to differentiate to chondrocytes and osteoblasts, respectively, in appropriate culture conditions, we observed differences in *ACVR1* expression levels that we interpret as referable to subtle and unintentional differences in standard culture conditions.

The most evident change, observed in all cell lines, was decreased activity upon deletion of the sequence between -762 and -308 bp. This region is characterized by a high degree of conservation and a number of binding sites for several transcription factors that were detected by ENCODE [20,34]. Our initial characterization of the effect of some of these transcription factors focused on those with possible implications for a FOP pathogenic mechanism. Their effect on the different promoter/reporter vectors varies according to the presence of putative binding sites in one or more than one position within the 2.9 kb region and also according to the cell type in which transfection experiments were performed.

The most consistent effect was by Hey-1 which showed inhibitory effects on promoter regions where E-box binding sites were predicted, i.e., in the region between -762 and -308 and in the basal 72 bp region immediately upstream of the TSS. The Hey-1 transcription factor is a member of the basic helix-loop-helix (bHLH) protein family and a known target of the Notch1 signaling pathway [27]. Interestingly, its expression is also regulated by SMAD-dependent BMP signaling that suggests involvement in cross-talk between these two important pathways [28,29]. Cross-talk is made more interesting by the observation that its effect on *ACVR1* expression could contribute to modulating the two pathways by a feed-back mechanism that acts on BMP signaling. The results of our co-transfection experiments are in agreement with several findings that support transcriptional inhibition of several target genes by Hey transcription factors [35].

Members of the "Early Growth Response" (Egr) family of transcription factors regulate gene transcription affecting a wide range of processes including differentiation, proliferation, and response to extracellular signals with cell type- and context-specific characteristics (see in [24]). The Egr-1 transcription factor, also known as Krox-24, is a zinc finger transcription factor originally identified as an early response gene that is rapidly stimulated by mitogenic and stress stimuli [36]. The specific DNA binding site for Egr-1 is GC-rich and present within the promoter regions of a large number of target genes important for proliferation, differentiation, apoptosis, growth control, and inflammation [24,37-40]. Egr-1 null mice showed impairment of endochondral bone repair [21,22]. In our studies, the effect of Egr-1 on the *ACVR1* promoter was clearly cell context-dependent. Egr-1 showed no effect on the 2.9 kb promoter sequence in ATDC5 cells and strong activation in HeLa cells. However, Egr-1 inhibited expression from the short 72 bp basal promoter sequence in U2OS, ATDC5 and C2C12 cells, with no effect in HeLa cells.

The ZBTB7A gene encodes the Leukemia/lymphoma-related factor (LRF, also called Pokemon, a member of the POK (POZ/BTB and Krüppel) family of transcriptional repressors [41]. This factor has been mainly studied for roles in several types of cancer [25] as well as in lymphocyte differentiation in the hematopoietic system [25,26]. ZBTB7A was also studied in the skeletal system as an osteoclast-specific protein [23]. ZBTB7A inhibited *ACVR1* in ATDC5 cells, which was an expected finding given many reports that describe its action as a transcriptional repressor. However, it was surprising to observe a strong activating effect in HeLa cells.

We found that a short sequence within the first 72 bp upstream of the *ACVR1* TSS, displayed readily detectable transcriptional activity. This region is a GC-rich sequence which, as in other TATA-less promoters like *ACVR1*, is associated with transcription initiation. As expected for a functionally important region, a high degree of conservation among species around the +1 TSS is observed.

Based on consensus sequences for conserved binding elements, the Sp1 transcription factor and other GC binding proteins were predicted to bind and affect the 72 bp region reporter activity.

Our transfection experiments support a role for Sp1, although Sp1 appears to induce different degrees of activation in the various cell lines examined. The highest response was in C2C12 cells, suggesting that the Sp1 transcription factor participates not only at the level of minimal promoter functionality but also in cell-type related functions. One possible explanation may be due to cooperative effects of Sp1 with other factors that bind GC-rich sequences. Among these factors, we observed a cell type activating effect on the *ACVR1* basal promoter by another member of the Egr family, the Egr-2/Krox20 transcription factor. This finding is of particular interest due to the implication of Egr-2 in many processes such as bone metabolism [42,43], immune response, inflammation and monocytic/macrophagic differentiation that are critical in FOP pathophysiology [42-44].

The base composition of the GC-rich 72 bp sequence raised the question of its possible ability to include a BMP responsive element [33] which is itself a GC-rich sequence. Our experiments did not show response to BMP2 by the 72 bp promoter region, although the 2.9 kb *ACVR1* promoter was activated by BMP2 treatment and can be attributed to the presence of BREs in regions upstream of the short minimal promoter.

Our results highlight cell-type specific functional features in the *ACVR1* gene regulation. Further investigations will be necessary to understand which transcriptional regulators play specific roles in various cell types and biological processes, as well as to also understand their utility in addressing new therapeutic approaches.

## Conclusions

The ultimate goal of research on a rare disease such as FOP is the development of a specific treatment that might efficiently prevent or even reverse the occurrence of disease flare-ups and their devastating consequences. Discovery of the causative gene represented a milestone in the process of understanding the genetic and molecular basis of FOP. Many approaches are currently being pursued to achieve proof of principle for developing specific therapeutic tools. Some strategies are aimed at specifically silencing the mutated allele, while others are aimed at identifying molecules that inhibit the activity of the protein, or to target downstream signaling (see for references and comments [2]) [45-48].

Any intervention able to restrain heterotopic bone formation and/or its inciting events will benefit patients’ quality of life. Therefore, the knowledge of mechanisms and factors that control gene function and regulation have great potential to add an important piece to the FOP pathogenesis puzzle.

The characterization of the *ACVR1* gene promoter has determined that this gene is potently regulated at the transcriptional level and that such regulation is significantly dependent on the cellular context. The identification of two essential regions of the *ACVR1* promoter suggests several hypotheses related to the FOP pathogenic mechanism. In particular, the cell types that are involved in the disease certainly include multipotent progenitors that can differentiate to chondrocytes and osteoblast but also include cells of the inflammatory and immune-mediated processes that trigger the acute phases of the disease and stimulate osteogenesis. In patients, each of these cell types carry the mutated receptor which is likely to exert effects at different steps of the pathogenic mechanism and then on the full FOP clinical picture. For this reason, we believe that this study is a good starting point to define the *ACVR1* gene regulation in different steps of the disease and to provide useful information on possible targets for potential treatment strategies.

## Competing interests

The authors declare that they have no competing interests.

## Authors’ contributions

FG conceived, designed, performed and analyzed the experiments and prepared the manuscript; SC prepared deletions plasmids and performed transfection experiments; LT and MM performed and analyzed RT-qPCR experiments; SDL performed and analyzed with DF EMSA experiments, RR and RB conceived and supervised the experiments and prepared the manuscript. All authors read and approved the final manuscript.

## Supplementary Material

Additional file 1: Figure S1 ENCODE (Encyclopedia of DNA Elements) data in the *ACVR1* promoter region. Track from the UCSC Genome Browser showing the ChIP-Seq data from the ENCODE Project (window coordinates, chr2:158732343-158735255) in the 2.9 kb genomic region of the *ACVR1* promoter. The black line with numbers (with the arrow specifying the orientation of the *ACVR1* gene), and the red vertical dashed lines have been added to indicate the positions of the reporter construct boundaries (bPr, basal 72-bp Promoter; Pr-0.3, Pr-0.7, Pr-1.2, Pr-1.4 and Pr-2.9). The localization patterns along the *ACVR1* promoter region of the different transcription factors listed on the right side are shown as rectangles of different color intensity. The transcription factors studied in this work are underlined. Click here for file

Additional file 2: Figure S2 Mithramycin effect on endogenous *ACVR*1 mRNA expression. *ACVR*1 mRNA expression level was evaluated by RT-qPCR in U2OS and C2C12 cells, both in basal conditions or upon treatment with mithramycin A at 200 (M200) and 300 nM (M300) final concentration. Values were normalized to the expression level of *GAPDH* and *β*-*Actin* genes. Error bars indicate the standard errors of three independent experiments. Click here for file

Additional file 3: Figure S3 Effects of Egr-1 and Egr-2 on basal promoter regulation. U2OS, HeLa, C2C12 and ATDC5 cells were co-transfected with the Pr-0.072 reporter construct and expression vectors carrying the Egr-1 (upper panel) or Egr-2 (bottom panel) cDNAs or the corresponding empty vector. Observed Luciferase activity is expressed as relative to the activity of the Pr-0.072 construct co-transfected with the empty vector (*ev*). The data represent the means ± SD (error bars) of three independent experiments carried out in triplicate with *p* < 0.05^*^, *p* < 0.01^**^, or *p*< 0.001***. Click here for file
