# Supplementary figures and images for: Identification and characterization of regulatory elements in the promoter of ACVR1, the gene mutated in Fibrodysplasia Ossificans Progressiva
Source: Orphanet J Rare Dis. 2013 Sep 18;8:145. doi: 10.1186/1750-1172-8-145 (PMC4015442; doi:10.1186/1750-1172-8-145)

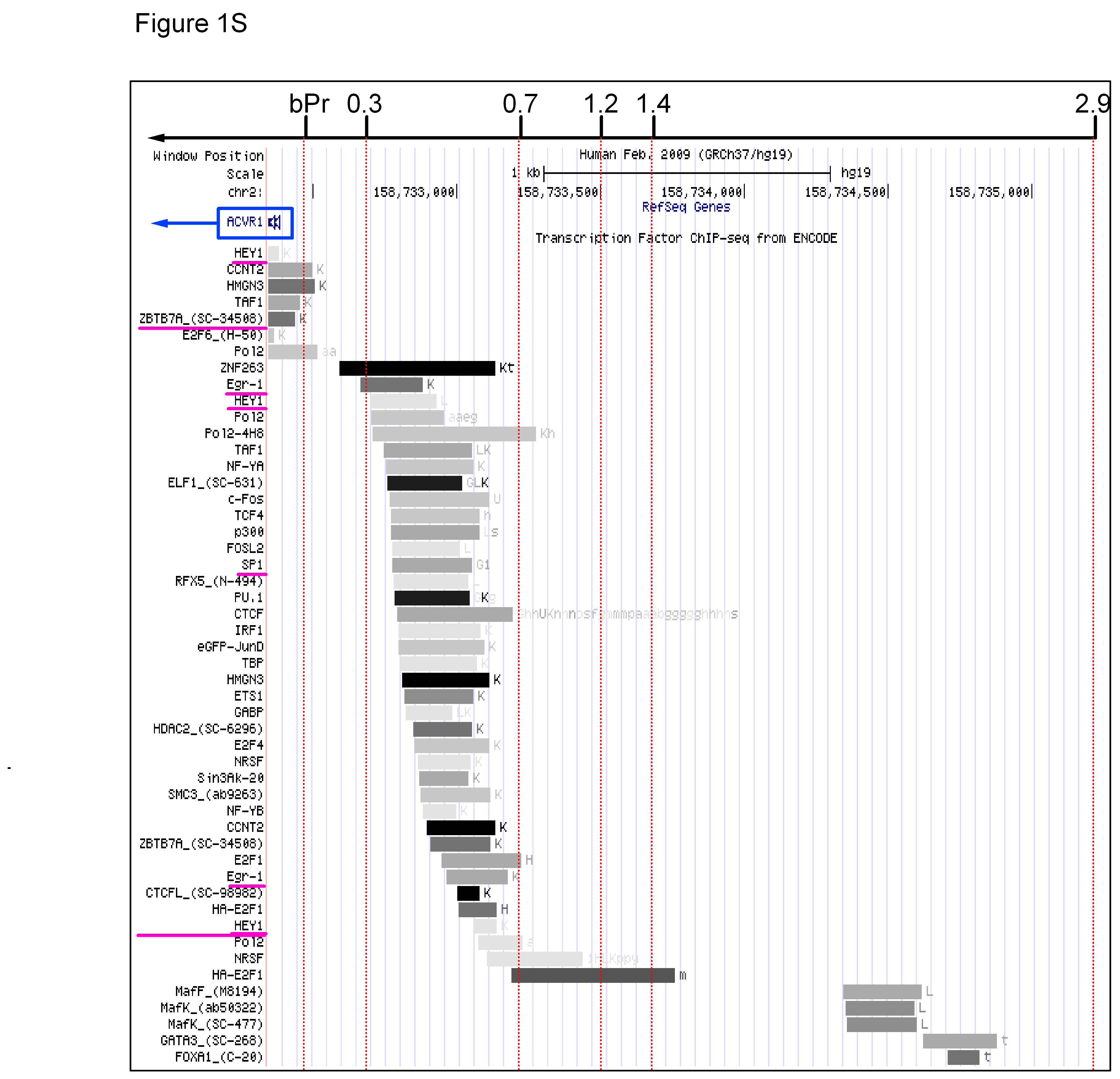

Supplement: Additional file 1: Figure S1 — ENCODE (Encyclopedia of DNA Elements) data in the ACVR1 promoter region. Track from the UCSC Genome Browser showing the ChIP-Seq data from the ENCODE Project (window coordinates, chr2:158732343-158735255) in the 2.9 kb genomic region of the ACVR1 promoter. The black line with numbers (with the arrow specifying the orientation of the ACVR1 gene), and the red vertical dashed lines have been added to indicate the positions of the reporter construct boundaries (bPr, basal 72-bp Promoter; Pr-0.3, Pr-0.7, Pr-1.2, Pr-1.4 and Pr-2.9). The localization patterns along the ACVR1 promoter region of the different transcription factors listed on the right side are shown as rectangles of different color intensity. The transcription factors studied in this work are underlined. [file 1750-1172-8-145-S1.tiff]

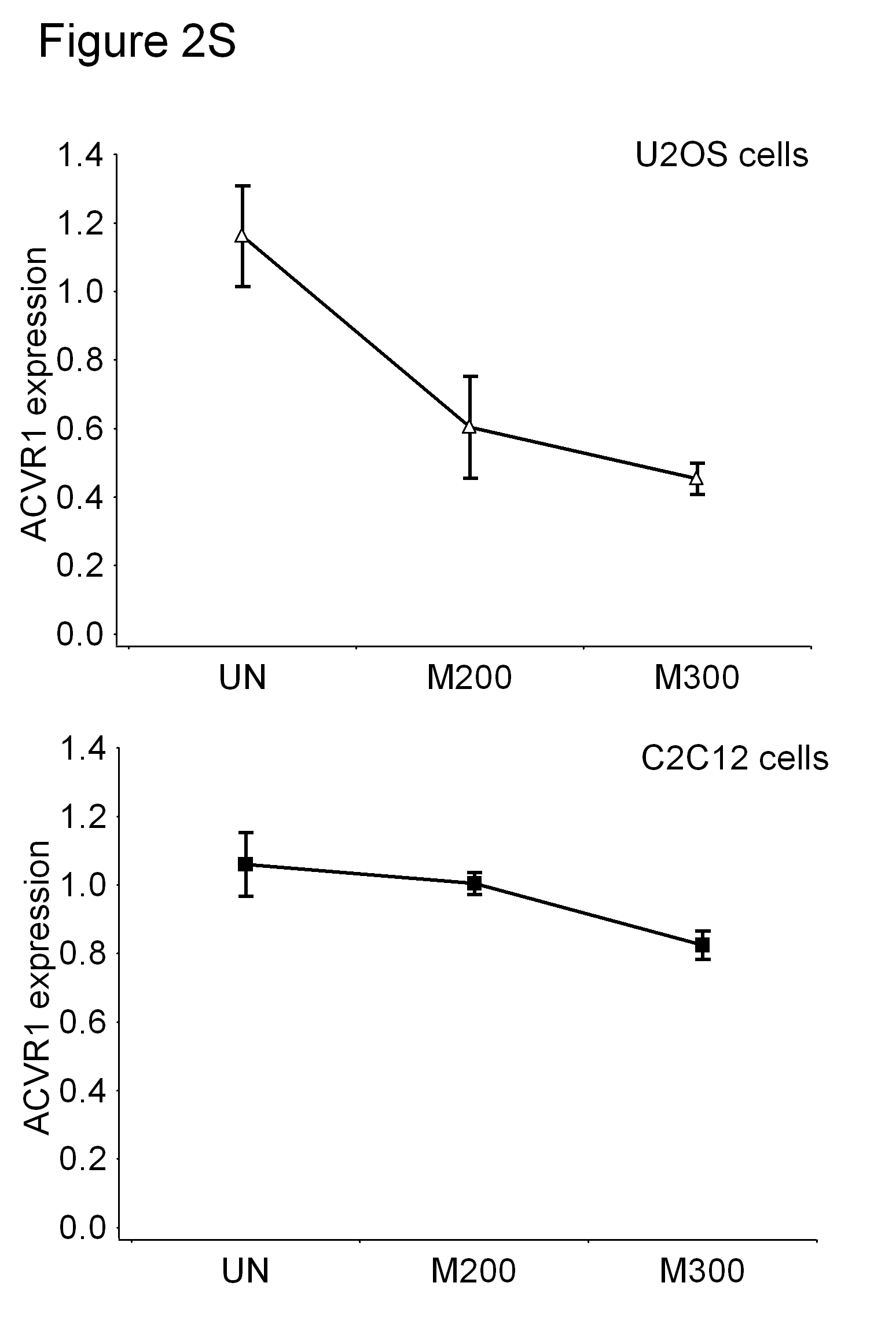

Supplement: Additional file 2: Figure S2 — Mithramycin effect on endogenous ACVR1 mRNA expression. ACVR1 mRNA expression level was evaluated by RT-qPCR in U2OS and C2C12 cells, both in basal conditions or upon treatment with mithramycin A at 200 (M200) and 300 nM (M300) final concentration. Values were normalized to the expression level of GAPDH and β-Actin genes. Error bars indicate the standard errors of three independent experiments. [file 1750-1172-8-145-S2.tiff]

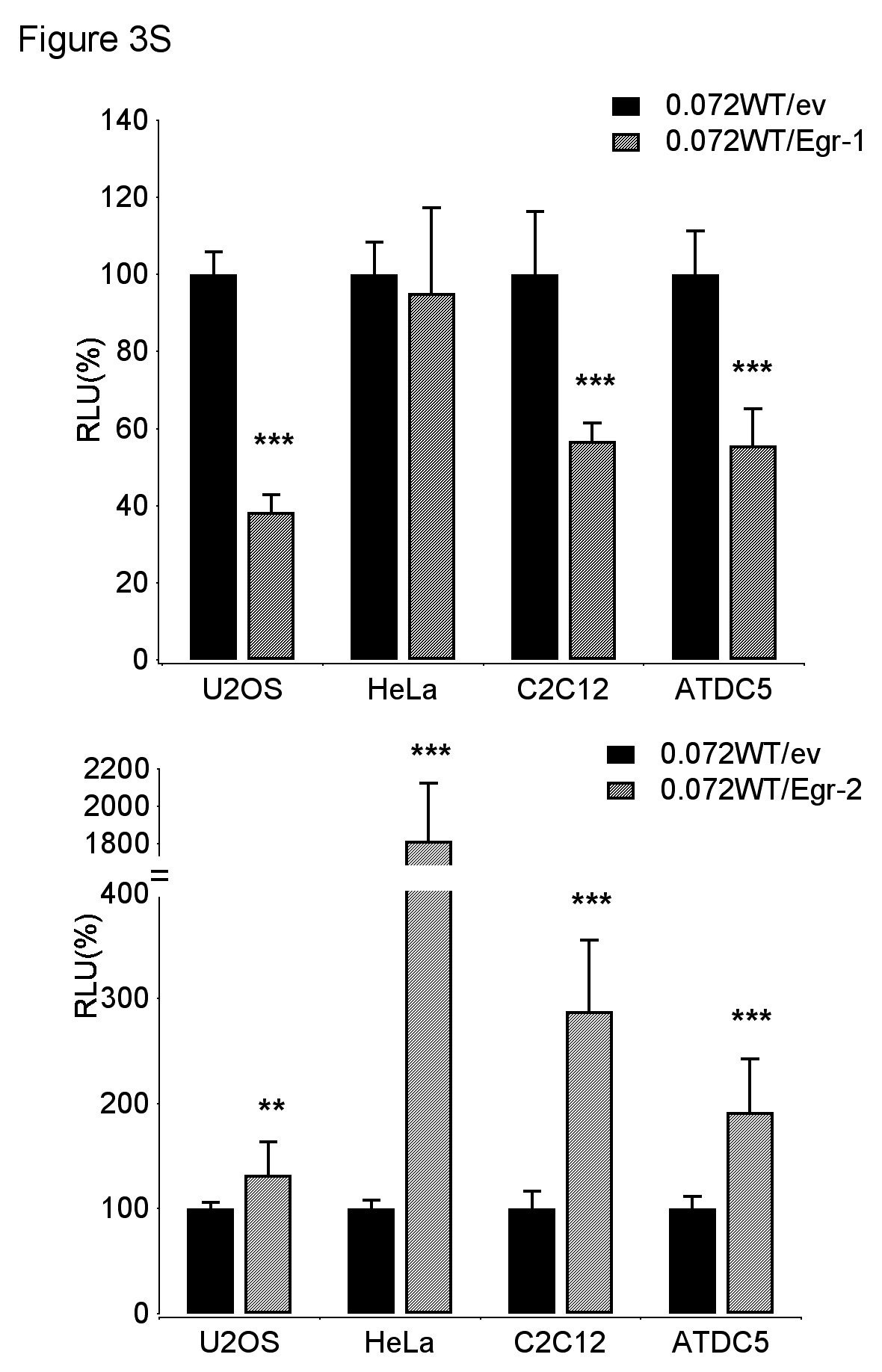

Supplement: Additional file 3: Figure S3 — Effects of Egr-1 and Egr-2 on basal promoter regulation. U2OS, HeLa, C2C12 and ATDC5 cells were co-transfected with the Pr-0.072 reporter construct and expression vectors carrying the Egr-1 (upper panel) or Egr-2 (bottom panel) cDNAs or the corresponding empty vector. Observed Luciferase activity is expressed as relative to the activity of the Pr-0.072 construct co-transfected with the empty vector (ev). The data represent the means ± SD (error bars) of three independent experiments carried out in triplicate with p < 0.05*, p < 0.01**, or p< 0.001***. [file 1750-1172-8-145-S3.jpeg]
